# Quantifying tumor associated macrophages in breast cancer: a comparison of iron and fluorine-based MRI cell tracking

**DOI:** 10.1038/srep42109

**Published:** 2017-02-08

**Authors:** Ashley V. Makela, Jeffrey M. Gaudet, Paula J. Foster

**Affiliations:** 1 Robarts Research Institute, London, Ontario, Canada; 2 The Department of Medical Biophysics, Western University, London, Ontario, Canada

## Abstract

Tumor associated macrophages (TAMs) are associated with tumor growth and metastasis. MRI can detect TAMs labeled with iron oxide (USPIO) or perfluorocarbon (PFC) agents. This study compared these two cell tracking approaches for imaging TAMs *in vivo*. 4T1 tumors were imaged with MRI at 4 days or 3 weeks post cell implantation after intravenous (i.v.) administration of either USPIO or PFC. Signal loss was detected within tumors at both time points post USPIO. Images acquired at 4 days demonstrated signal loss encompassing the entire tumor and around the periphery at 3 weeks. Number of black voxels suggested higher numbers of TAMs in the tumor at the later time point. After PFC administration, Fluorine-19 (^19^F) signal was detected in a similar spatial distribution as signal loss post USPIO. ^19^F signal quantification revealed that the number of ^19^F spins was not significantly different at the two time points, suggesting a similar number of TAMs were present in tumors but accumulated in different regions. ^19^F signal was higher centrally in tumors at 4 days and heterogenous around the periphery at 3 weeks. This study revealed that ^19^F-based cell tracking methods better represent TAM density and provides additional information not achievable with iron-based methods.

Inflammation is a key determinant in cancer behavior, playing a pivotal role in cancer initiation and progression^1^. Tumor-associated macrophages (TAMs) are the major leukocyte population among the tumor-infiltrating immune cells. Macrophages have been shown to play a dual role in tumor growth either driving tumor rejection or tumor progression, depending on timing and type of macrophage activation^2^.

In breast cancer, TAMs have been linked to increased metastasis, angiogenesis and tumor aggressiveness^2^,^3^. Histological studies of biopsies from human breast tumors showed significantly higher numbers of TAMs in malignant tumors compared with benign tumors^4^. More recent studies of breast cancer patients show that the presence of CD163+ macrophages (TAMs) positively correlates with triple negative breast cancer (TNBC)^5^. Therefore, recruitment of TAMs could play a significant role in the outcome of TNBC patients and explain their poor prognosis. Experimental evidence also suggests that TAMs influence the metastatic potential of tumors^2^. Overall, high infiltration of TAMs in breast tumors predicts unfavorable outcomes.

Most studies of TAMs in breast cancer are performed using histology; a number of these approaches have been reviewed^6^. Although these methods are able to provide detailed molecular and morphologic information, they are limited by the need to euthanize the animal. This permits only an end point analysis and limited amounts of sectioning, so that only a small portion of a tumor is typically interrogated. The ability to observe TAMs in breast cancer non-invasively and longitudinally *in vivo* would add tremendously to our understanding of the spatial and temporal changes of this cell population in the tumor microenvironment.

Macrophages can be imaged using *in vivo* cellular MRI techniques. To do this, iron nanoparticles are administered intravenously (i.v.) and are subsequently internalized by monocytes/macrophages in the reticuloendothelial system (specifically, the Kupffer cells of the liver and macrophages in the spleen, bone marrow and lymph nodes). In the presence of an inflammatory condition, iron nanoparticles are taken up by associated macrophages and accumulate in the affected organ/tissue. MRI is typically performed one day after the administration of iron, to allow time for the accumulation of labeled cells and to permit clearance of intravascular iron^7^. The *in vivo* labeling efficiency of macrophages is dependent upon many factors including macrophage polarization and the type of nanoparticle used (amount, size, coating); not all macrophages become labeled. However, most of these studies use ultra-small superparamagnetic iron oxide (USPIO) agents, which are smaller and have longer circulation times compared to larger iron oxide agents, leading to greater uptake. Areas which contain iron-labeled inflammatory cells appear as regions of low signal intensity in iron sensitive MR images. This approach has been used to image inflammation in many diseases including atherosclerosis^8^, multiple sclerosis^9^ and arthritis^10^. TAMs have been previously imaged this way in a preclinical models of breast^7^,^11^, lung^12^, colon^12^, hepatic^13^ and nasopharyngeal^14^ cancer. Cellular MRI has high sensitivity for the detection of iron-labeled TAMs, but is limited by low specificity. In addition, quantification of the signal loss caused by iron-labeled cells is complicated due to the non-linear relationship between iron concentration and signal loss.

The use of a fluorine-19 (^19^F) imaging agent is a newly emerging technique for MRI cell tracking. ^19^F-based cell tracking of macrophages is achieved through the administration of perfluorocarbons (PFCs), which are phagocytosed *in vivo* by macrophages, similar to the uptake of iron nanoparticles previously described. ^19^F MRI addresses some of the limitations associated with iron-labeled cell tracking. First, there is no MRI detectable fluorine in the native tissue, which allows for high specificity. Second, in contrast to the indirect visualization of iron-labeled cells by observed signal loss, the spins of ^19^F nuclei are directly detected and ^19^F signal is proportional to the number of ^19^F spins per voxel.

The main limitation of ^19^F MRI cell tracking is lower sensitivity. For ^19^F MRI to produce image quality similar to that of proton images used to detect signal loss due to iron, there must be a very high concentration of ^19^F atoms. Most studies have been performed at higher magnetic field strengths since low signal to noise ratios (SNR) present challenges due to the low ^19^F concentrations *in vivo*. The current cellular detection sensitivity for ^19^F MRI is estimated to be on the order of thousands of ^19^F labeled cells per voxel. Where there are lower numbers of labeled cells, they may not be detected and therefore there is a risk of underestimating cell number. ^19^F-based cell tracking methods have been applied to detect and monitor inflammation in a variety of experimental models including arthritis^15^, cancer^16^,^17^, pulmonary inflammation^18^, multiple sclerosis^19^, cardiovascular inflammation^20^ and transplantation rejection models^21^,^22^. This method has been reviewed by a number of groups^23^,^24^,^25^. In this paper we compare iron-based and ^19^F-based MRI for the identification and quantification of TAMs in a mouse model of breast cancer.

## Materials and Methods

### Cell Culture

4T1 murine breast cancer cells (Dr. Fred Miller, Wayne State University, MI, USA) were chosen due to their known TAM content^26^. This cell line closely resembles a human, late stage breast cancer which exhibits metastases^27^. Cells were maintained at 37 °C and 5% CO_2_ in Dulbecco’s Modified Eagle’s Medium-high glucose media (Invitrogen, Ontario, Canada) supplemented with 10% fetal bovine serum and passaged every 2–3 days.

### Animal Model

Female BALB/c mice (6–7 weeks; Charles River Canada) were obtained and cared for in accordance with the standards of the Canadian Council on Animal Care, under an approved protocol by the Animal Use Subcommittee of Western University’s Council on Animal Care. Mice (n = 20) were anesthetized with isoflurane administered at 2% in oxygen followed by an injection of 300,000 4T1 cells (>95% viability, measured using the trypan blue exclusion assay) suspended in 50 μl Hanks balanced salt solution (HBSS) into the 4^th^ mammary fat pad. The injection site was monitored daily. For iron-based imaging, 0.5 mmol/kg Ferahame (Ferumoxytol, AMAG Pharmaceuticals Inc, MA, USA) was injected via tail vein 24 hours before post iron imaging. For fluorine-based imaging, mice were administered 200 μl (~7.8E19 ^19^F atoms in 1 ml) PFC agent (V-Sense, VS-1000H DM Red; CelSense Inc. Pittsburgh, USA) via the tail vein 48 hours prior to imaging. This PFC has a red fluorescent tag (Texas Red). Animals were observed until alert and active, when they were returned to their cages.

### MRI

*In vivo* proton (^1^H) and ^19^F MRI were performed at two time points: (1) when the tumor was first palpable (imaged at 4 days) and (2) at 3 weeks post cancer cell implantation. While imaging, mice were anesthetized with 2% isoflurane in oxygen. *USPIO imaging (4 day n = 4, 3 week n = 7*): Images were obtained pre and 24 hours post i.v. USPIO on a 3 Tesla (T) GE clinical MR system (General Electric, Mississauga, Canada) equipped with a custom built insertable gradient coil (maximum gradient strength 500 mT/m and peak slew rate 3000 T/m/s) and using a solenoidal mouse body radiofrequency (RF) coil. A 3D balanced steady state free precession (bSSFP) imaging sequence was used to obtain high resolution ^1^H images of the whole mouse body. Image resolution was 200 × 200 × 200 μm and sequence parameters were as follows: FOV = 60 × 30 mm, matrix = 300 × 150, flip angle = 35°, bandwidth = 31 kHz, TR/TE = 6.3/3.1 ms, 2 averages and 8 phase cycles resulting in a scan time of ~30 minutes. *PFC imaging (4 day n = 5, 3 week n = 4*): ^1^H and ^19^F images were acquired 48 hours post PFC with a 9.4 T Varian small animal MRI system using a custom built dual ^1^H/^19^F birdcage coil, tuned to 400.2 MHz for proton and 376.8 MHz for ^19^F imaging. A higher magnetic field was used for ^19^F imaging due to the lower detection sensitivity. Reference tubes of known ^19^F concentration (3.33 × 10^16 19^F/μl) were placed alongside the mouse for quantification purposes. ^1^H and ^19^F images were acquired with a bSSFP sequence. ^1^H images were acquired with 200 × 200 × 200 μm spatial resolution and the following parameters: FOV = 50 × 36 × 36 mm, matrix = 250 × 180 × 180, flip angle = 30°, bandwidth = 87 kHz, TR/TE = 3.9/1.9 ms, 3 averages and 4 phase cycles with a scan time of ~20 minutes. ^19^F images were acquired with 500 × 500 × 1000 μm spatial resolution with the following parameters: FOV = 50 × 36 × 36 mm, matrix = 100 × 72 × 36, flip angle = 70°, bandwidth = 25 kHz, TR/TE = 5.5/2.8 ms, 30 averages and 4 phase cycles resulting in a scan time of ~35 minutes. Excitation was performed with a 1 kHz gauss pulse to prevent detection of isoflurane.

### Histological Analysis

Mice were euthanized via intraperitoneal injection of sodium pentobarbital (Euthanyl;Bimeda-MTC Animal Health Inc., Cambridge, ON, Canada) immediately following the last MR exam. The tumors from mice that received USPIO were excised and placed in 4% formalin for fixation. Tumors were paraffin embedded, sectioned (6 μm) and subsequently stained with hematoxylin and eosin (H&E), Perls’ Prussian Blue (PPB) for iron and biotinylated isolectin B4 (Sigma Adrich) which binds to the galactose-type terminal present on activated macrophages. Mice that received PFC were transcardially perfused with saline followed by 4% paraformaldehyde (PFA) to reduce autofluorescence within specimens. Tumors were excised and cryoprotected by passage through a sucrose gradient of 10%, 20% and 30% for 24 hours each. Samples were then frozen in an OCT compound and cryostat sections were collected (10 μm). Diaminobenzidine (DAB) and fluorescent immunohistochemistry (IHC) was performed for lectin and fluorescent images were collected to detect the red-fluorescence from the PFC agent.

Microscopy was performed using a Zeiss Axio Imager A1 microscope (Zeiss Canada, Toronto, ON, Canada) equipped with a Retiga EXi (QImaging Scientific Research Cameras, Surrey, BC, Canada) digital camera. The use of a slide scanner (TissueScope, Huron Technologies, Waterloo, ON, Canada) allowed for digital whole tumor section scanning (0.5 μm resolution).

### Analysis of MRI data

#### USPIO imaging

Analysis of signal loss in tumor images was performed using OsiriX imaging software (Pixmeo SARL, Bernex, Switzerland). Pre and post iron images were compared to detect regions of signal loss that were present within tumors post USPIO. Tumors were manually outlined slice by slice in post iron images, creating a 3D tumor volume. The total number of black voxels per tumor (defined as the voxels which contain signal loss) was then determined from these volumes by setting a threshold. The threshold was chosen by measuring the mean signal intensity +2 standard deviations from within the signal void region. Then the total number of black voxels with signal intensity values below this threshold value was obtained from the tumor histogram. The 3D isotropic voxels allowed multi-planar reformatting to compare the spatial distribution of PPB staining of a whole tumor section to signal loss on MR images. *PFC Imaging:*^19^F images were overlaid onto the proton images to determine where the ^19^F signal was located within the mouse. From ^19^F images, voxels containing ^19^F signal which were within the tumor were manually delineated and ^19^F quantification was performed using VoxelTracker software (Celsense Inc., Pittsburgh, Philadelphia, USA). In brief, the total number of ^19^F spins were determined by comparing the sum of the ^19^F signal within a region of interest to the signal generated by reference tubes containing a known amount of ^19^F spins (3.33 × 10^16 19^F/μl) and taking into consideration signal from noise.

### Statistical Analysis

Statistical analyses were performed using GraphPad Prism software (GraphPad Software Inc, La Jolla, CA, USA). Data are expressed as mean ± SD. Unpaired, two-tailed t-tests were performed on quantification between time points, within imaging method; *p* < 0.05 was considered a significant finding.

## Results

**Figure 1:**
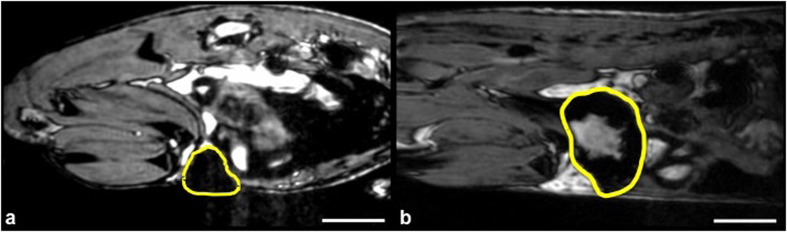
Iron-based MRI cell tracking. Sagittal bSSFP images of mammary fat pad tumors acquired 24 hours post USPIO administration. (**a**) 4 days post 4T1 cancer cell injection: Signal loss encompasses the entire tumor volume (**b**) 3 weeks post 4T1 cancer cell injection: The tumor is larger; signal loss is now seen around the periphery of the tumor and not within the central core. Scale bars represent 5 mm.

All mice developed 4T1 mammary fat pad tumors. Images of mice that received USPIO showed regions of signal hypointensity within the tumors. For mice that were injected on day 3 post cell implantation and imaged on day 4, tumors were relatively small; the mean tumor volume was 26.7 mm^3^ and the region of signal loss covered the entire tumor volume. For mice imaged at 3 weeks post cell implantation the regions of signal loss were mainly in the periphery and not within the central region of the tumor. Tumors were significantly larger at this stage; the mean tumor volume was 534 mm^3^. Representative images acquired post USPIO are shown in Fig 1.

**Figure 2:**
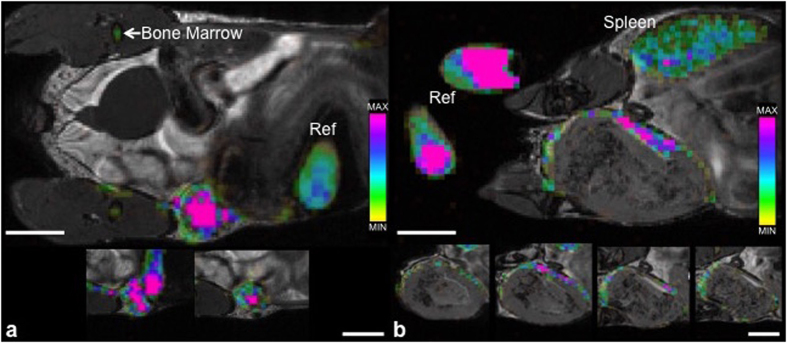
^19^F-based MRI cell tracking. bSSFP images of mammary fat pad tumors acquired 48 hours post PFC administration. (**a**) 4 days post 4T1 cancer cell injection: ^19^F signal is detected throughout the entire tumor with a higher density visualized within the center of the tumor (pink) when compared to the periphery. (**b**) 3 weeks post 4T1 cancer cell injection: ^19^F signal is detected, heterogeneous in density, only along the periphery of the tumor. Cropped images that are placed below each of the main images show the adjacent image slices (1 mm) containing ^19^F signal. The color bar demonstrates range of ^19^F spins. ^19^F signal is also detected in reference tubes (ref), bone marrow and spleen. Scale bars represent 5 mm.

^19^F MRI images were created by an overlay of hydrogen (^1^H) and ^19^F MR datasets (Supplementary Figure S1) which were obtained post PFC; these images showed ^19^F signal in a pattern similar to the post USPIO images for each time point. The number of ^19^F spins per voxel can be determined from these images, allowing for mapping of the ^19^F density over the tumor volume. Fig. 2 shows ^19^F images at both time points, with adjacent slices below demonstrating signal distribution through the tumor. For mice that were imaged on day 4 there is ^19^F signal throughout the whole tumor, with a higher number of ^19^F spins in the central region of the tumor compared to the periphery (Fig. 2a). For mice imaged at 3 weeks the ^19^F signal is around the periphery and the number of ^19^F spins is heterogeneous in this region (Fig. 2b).

**Figure 3:**
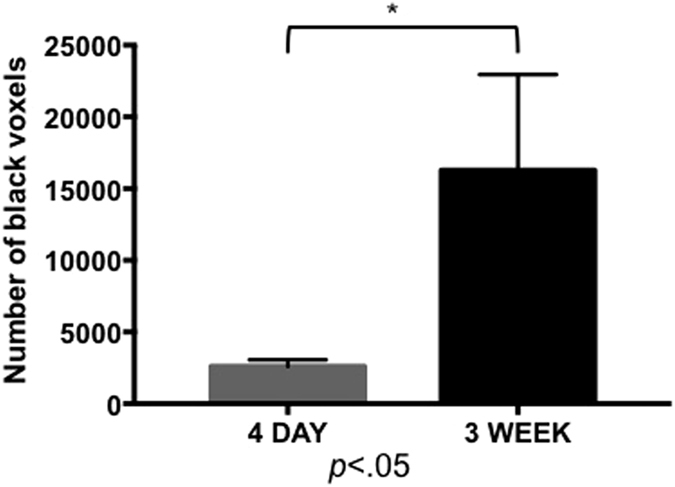
USPIO-based MRI quantification. Signal loss from iron was quantified by measuring the number of black voxels in whole tumor volumes. Mean values with standard deviation are shown for each time point. The asterisk denotes a significantly higher number of black voxels (*p < 0.05; *t-test*) were detected at 3 weeks (n = 7) compared to 4 days (n = 4).

**Figure 4:**
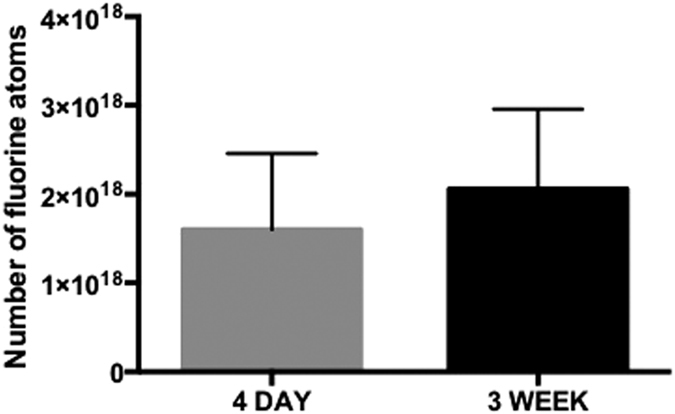
PFC-based MRI quantification. Quantification of the number of ^19^F spins was achieved by manually selecting voxels with ^19^F signal within the whole tumor. Mean values with standard deviation are shown for each time point. No significant difference (*p* > 0.05; *t-test*) was found between the number of ^19^F spins in whole tumor volumes between 4 day (n = 5) and 3 week (n = 4) post 4T1 cancer cell injection.

For post USPIO images the signal loss was quantified by measuring the number of black voxels for each tumor volume, presented as mean values and standard deviation for each time point (Fig. 3). This analysis suggests that there is a significant increase (*p* = 0.003) in the number of TAMs at 3 weeks compared to 4 days. For post PFC imaging the number of ^19^F spins was determined for each tumor volume, presented as mean values and standard deviation for each time point (Fig. 4). Analysis of these results indicates that there is no significant difference (*p* = 0.4614) in TAMs between the two time points.

**Figure 5:**
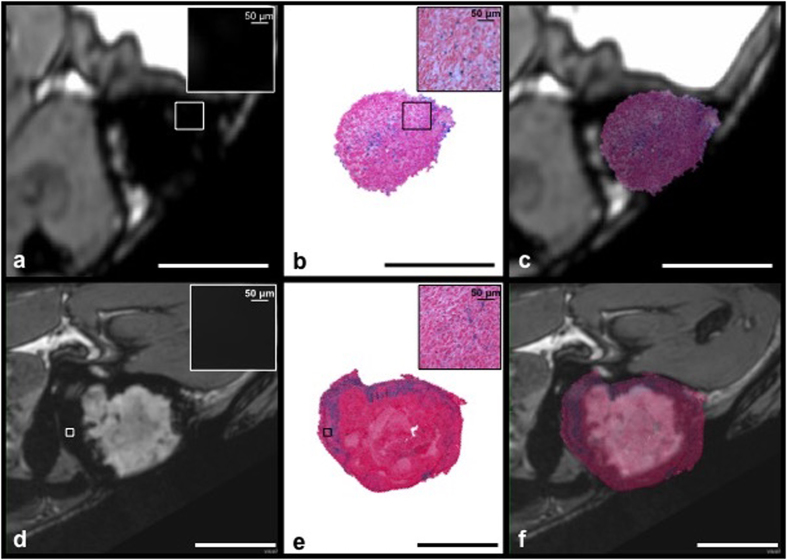
Good spatial correspondence between signal loss on MRI and PPB stained whole tumor sections. MRI acquired 24 hours post i.v. iron, the corresponding PPB stained tumor sections and PPB staining overlaid onto the images. (**a**–**c**) 4 days post 4T1 cancer cell injection and (**d**–**f**) 3 weeks post 4T1 cancer cell injection. The spatial distribution of PPB stained cells relates well to where signal loss is observed by MRI although it is clear that the blooming effect of iron in proton images leads to a greater area of signal void than is actually occupied by the iron-positive cells. This is demonstrated clearly by looking at the higher magnification views of regions of signal loss (insets, a,d) which show that there is total signal loss due to USPIO-labeled cells and the corresponding high magnification views of PPB staining in these areas (insets, b,e) which demonstrate that not all of the cells are PPB positive. Scale bars represent 5 mm.

PPB staining for iron in tumors showed good spatial correspondence with MRI. Representative PPB staining for tumors at each time point is shown in Fig. 5. In tumors examined post USPIO at 4 days, PPB-positive cells were observed scattered throughout the tumor (Fig. 5b). In contrast, PPB staining was primarily observed in the tumor periphery for the tumors examined at 3 weeks (Fig. 5e). Higher power magnification of the tumor regions where PPB staining was observed shows that not all cells in these regions are iron-positive (Fig. 5b and e, insets). MR images are zoomed in to demonstrate the blooming artifact (black) caused by iron nanoparticles, which leads to an overestimation of the space occupied by iron-labeled cells (Fig. 5a and d, insets).

**Figure 6:**
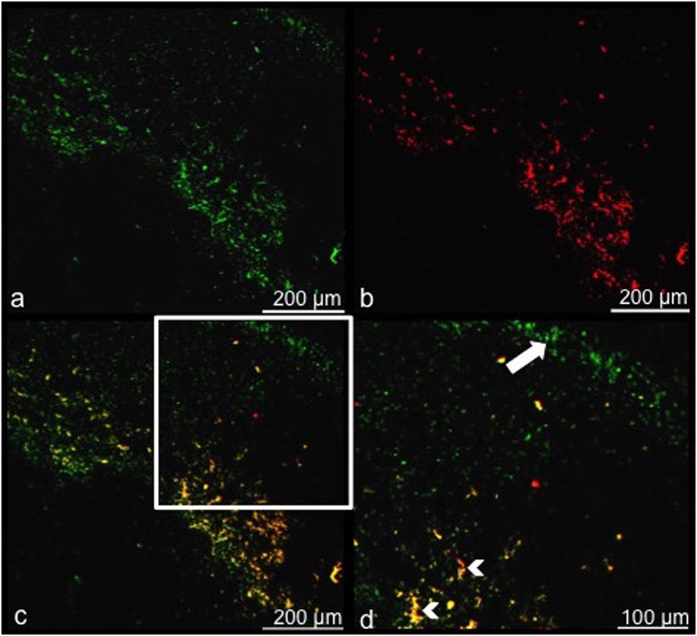
Fluorescence microscopy validates PFC labeled TAMs. Fluorescence microscopy visualizing the periphery of a tumor 3 weeks post 4T1 cancer cell injection validates that TAMs have been labeled *in vivo* with the PFC agent. (**a**) Lectin stained (activated) macrophages = green and (**b**) PFC agent = red. (**c**) An overlay demonstrates yellow PFC-labeled macrophages. (**d**) A zoomed in portion of the overlay shows the presence of both PFC-labeled macrophages (white arrowheads) and unlabeled macrophages (white arrow).

^19^F-based MRI signal detection was validated using fluorescence microscopy for macrophage staining and detection of the red fluorescent PFC. Fig. 6 shows the detection of positive lectin staining for macrophages (Fig. 6a, green) and red fluorescence associated with V-Sense (Fig. 6b) located in the periphery of tumor sections obtained at 3 weeks post implantation. When an overlay of these images is created, PFC labeled macrophages can be detected as yellow (white arrow heads). Note there are also macrophages that are not PFC labeled (Fig. 6d, white arrow) – just green – this is to be expected since PFC is administered 2 days prior to MRI and the removal of tumors and TAM infiltration is dynamic.

## Discussion

In this study we have assessed iron-based and ^19^F-based MRI methods for imaging TAMs. To the best of our knowledge, this is the first time these two imaging approaches have been compared for TAM detection in the same cancer model. Two different MRI systems were utilized for these methods. Proton images for detection of signal loss due to iron-labeled cells were acquired using a 3 T clinical MRI system along with a custom built insertable gradient coil which permits high resolution imaging of mice on a human sized scanner. ^19^F images were acquired using a 9.4 T dedicated small animal MRI system to boost SNR as a way to compensate for decreased detection sensitivity.

Both imaging methods revealed the early presence of TAMs, when tumors were first palpable. Although the dynamics of immune cell infiltration in tumors is not well understood, several histology-based studies have observed a pronounced immune cell infiltration in very early, low-grade pre-invasive tumors. Clark *et al*. showed that TAMs, myeloid derived suppressor cells (MDSC) and regulatory T cells dominated the early response, and persisted as tumors became more invasive, in a model of pancreatic ductal carcinoma^28^. Effector T cells, on the other hand, were scarce in pre-invasive lesions and found only in advanced cancer. Their results suggest that immunosuppressive cells of the immune system may appear very early in tumor development. TAMs may initially arrive at the site of a developing tumor with a suppressive (M1) phenotype and soon after be converted to the tumor promoting (M2) type macrophage^2^. Extensive infiltration of TAMs was shown at one week after a tumor challenge in a humanized mouse model of prostate cancer. These TAMs were of mixed M1/M2 phenotype and simultaneously produced pro-inflammatory and anti-inflammatory cytokines^29^.

Both imaging methods we used showed the same spatial distribution of TAMs at each time point; throughout the entire tumor at 4 days and restricted to the periphery at 3 weeks. Past studies have used both iron and ^19^F-based cellular MRI methods to visualize TAMs in various cancer models and at various time points. One study injected iron nanoparticles on day 5 and used MRI to follow TAMs during growth of implanted tumor fragments^14^. Expansion of the tumor was accompanied by signal loss, confirmed to be related to TAM accumulation, and was specifically associated with the region of the implant which produced a budding tumor mass. Others have observed signal loss, attributed to TAMs, distributed heterogeneously throughout the tumor^7^,^11^. At this time only two studies have used ^19^F MRI to image macrophages associated with cancer. In a pilot study which detected ^19^F signal in a 4T1 tumor at one week post cancer cell injection, ^19^F signal due to macrophage uptake was observed within the outer margin of the tumor, thought to be contributing to the invasive front of the tumor^17^. In another study early macrophage recruitment was visualized in the tumor rim after administration of a PFC agent in Vaccinia virus colonized tumors^16^. The different patterns of signal loss in these studies is likely related to differences in TAM content which should be expected at different time points during tumorigenesis and for tumors with different levels of aggressiveness.

The anti-tumor or pro-tumor activity of TAMs may depend on their localization within cancer tissue^30^. Multiphoton intravital imaging techniques have been instrumental in showing that in well developed tumors TAMs are mainly localized in the peripheral tumor stroma and decrease in number towards the center^31^,^32^. The microenvironment at the tumor periphery is very different from that in the core. Hypoxia is associated with the center of a tumor. The tumor edge is thought of as a meeting place where recruited immune and stromal cells interact are located^33^ and acts as a highway for cell migration^34^. In mammary tumors the stroma of the tumor margin is filled with collagen fibers and TAMs found in the tumor margin have been shown to accumulate in and migrate within collagen rich regions^35^. TAMs are known to promote invasion of tumor cells at the tumor periphery by supplying pro-migratory factors such as epidermal growth factor (EGF), by regulating the production of collagen to accelerate tumor motility, and by promoting extracellular matrix proteolytic remodeling^33^.

Quantification of the signal measured by each imaging technique produced different results. The quantification of ^19^F signal, attributed to PFC-positive TAMs, indicated that the total number of ^19^F spins was not different in the small, early tumors compared to the larger tumors imaged at 3 weeks post cell injection. This result suggests the number of TAMs doesn’t change much during tumor development over this time period but that the location of the TAMs is altered. An *in vivo* murine study of TAMs has suggested they have a long life span (ie. at least 4–5 weeks) and although there is likely some turnover, this may be offset by local proliferation and monocyte infiltration^36^.

Unlike iron particles, which are detected through their indirect effects on the surrounding water protons, the ^19^F agent functions like a tracer in that ^19^F MRI directly detects the ^19^F nuclei that are associated with the labeled cells; the ^19^F signal is directly proportional to the number of ^19^F spins. Maps of the number of ^19^F spins in tumors provided additional information on TAM distribution, revealing a higher number of ^19^F spins (pink) in the central region of tumors imaged at 4 days post cell injection and a heterogeneous pattern of ^19^F spins around the periphery of tumors imaged at 3 weeks. We believe these methods will allow for longitudinal monitoring of the density and distribution of TAMs in the same subjects, which could provide valuable information on TAM localization and accumulation over time.

The quantification of signal loss, attributed to iron-positive TAMs, suggested that TAM infiltration was greater in larger tumors imaged at 3 weeks post cell implantation compared to early tumors imaged at day 4. Quantifying signal loss, however, is not straightforward because the relationship between signal loss and iron concentration is not linear and because the presence of iron produces a localized disruption to the magnetic field homogeneity, resulting in a region of signal loss that extends far beyond the actual boundary of the cells. This is a susceptibility related artifact, known as the blooming effect^37^. The size of the blooming artifact depends on several factors including: magnetic field strength, type of iron particle, amount of iron particle uptake by the cell, the pulse sequence and pulse sequence parameters^37^. In bSSFP images the blooming artifact is relatively small, compared to standard gradient echo images which are more commonly used, but it can still be in the range of 20–50 times the size of the cell^38^. Every USPIO positive cell within the tumor will contribute to the signal loss within the tumor and depending on the size of the signal void, signal loss from multiple cells will overlap and will cause complete signal loss in areas of accumulation. For quantification, we chose a volumetric measure, the number of black voxels per tumor. Brisset *et al*. compared four quantitative methods for quantification of iron-labeled cells transplanted into mouse brain: (1) T2 relaxometry, (2) T2* relaxometry, (3) the volume of the cloverleaf hypointense artifact generated on T2*-weighted images, and (4) the volume of the cloverleaf hyperintense artifact generated on positive contrast images^39^. Only the volume of the cloverleaf artifact, whether measured from negative or positive contrast, correlated with cell number. Our histology showed that the regions of the tumor that contained iron-positive TAMs also contained other cells and iron-negative TAMs. Still, the blooming effect caused by the iron-positive TAMs encompassed the entire region (the whole tumor at day 4 and the entire periphery of the tumor at 3 weeks). This is important when considering the total area of signal loss. The iron-labeled cells are more dispersed throughout the larger tumor, and therefore lead to a calculation of a larger number of black voxels at the later time point; the signal loss covers more space in a larger area. In addition, when multiple nearby voxels contain iron-labeled cells the area of signal void produced by the blooming artifact may overlap. This highlights the challenges faced when measuring signal loss due to iron-labeled cells.

Other studies have used relaxometry to quantify signal loss due to iron-labeled cells. With multi-gradient echo imaging T2 or T2* relaxation can be measured and T2/T2* maps generated to show the distribution of iron-labeled cells in tumors. Although relaxometry data allows for a direct estimate of cell number, these studies have only shown fair agreement between the number of injected cells and the number estimated from T2 quantification and *in vitro* calibration curves^40^. In addition, the high iron content associated with accumulation of TAMs leads to very short T2/T2* decay which is too short to be accurately measured with standard gradient echo imaging. Ultra-short TE relaxometry approaches have been proposed as ways to improve quantification of iron-labeled cells^41^.

^19^F-based cell tracking does have some limitations when imaging TAMs. Compared to iron-based methods, the detection sensitivity for labeled cells is much lower for ^19^F. ^19^F MRI cell tracking likely underestimates the cell density in the tumor because of the low detection sensitivity; thousands of cells per voxel are required for detection of PFC-labeled cells, whereas single iron-labeled cells can be detected^37^,^42^. In experiments such as this one where the PFC agent is administered i.v., it is not possible to quantify the number of ^19^F labeled cells, only the number of ^19^F spins within the ROI. Quantification of cell number can only be determined in experiments where PFC is used to pre-label cells prior to implantation and the number of ^19^F spins per cell is known. Additional information can be determined post PFC administration if the tissues are removed and analyzed by ^19^F NMR. In this case, the total ^19^F content of the sample is determined and results normalized to tissue weight generating an “inflammation index”, expressed as the number of ^19^F spins per gram of tissue^19^.

Understanding the trafficking and timing of TAM infiltration will provide valuable insight into potential obstacles for developing anti-inflammatory treatments targeted against TAM recruitment and function that may be beneficial for both tumor prevention and therapy. ^19^F-based imaging of TAMs in individual breast tumors would allow for identification of breast tumors which are heavily infiltrated with TAMs and could be used as a biomarker for decisions about how to best treat these patients and for monitoring responses to therapy.

## Additional Information

**How to cite this article**: Makela, A. V. *et al*. Quantifying tumor associated macrophages in breast cancer: a comparison of iron and fluorine-based MRI cell tracking. *Sci. Rep.* **7**, 42109; doi: 10.1038/srep42109 (2017).

**Publisher's note:** Springer Nature remains neutral with regard to jurisdictional claims in published maps and institutional affiliations.

## Supplementary Material

Supplementary Figure S1
