# Supplementary material for: Quantifying tumor associated macrophages in breast cancer: a comparison of iron and fluorine-based MRI cell tracking
Source: Sci Rep. 2017 Feb 8;7:42109. doi: 10.1038/srep42109 (PMC5296729; doi:10.1038/srep42109)
Supplement: Supplementary Figure S1 [file srep42109-s1.pdf]

Quantifying tumor associated macrophages in breast cancer: a comparison of iron and fluorine-based MRI cell tracking

Ashley V. Makela*[1,2], Jeffrey M Gaudet[1,2], Paula J. Foster[1,2]

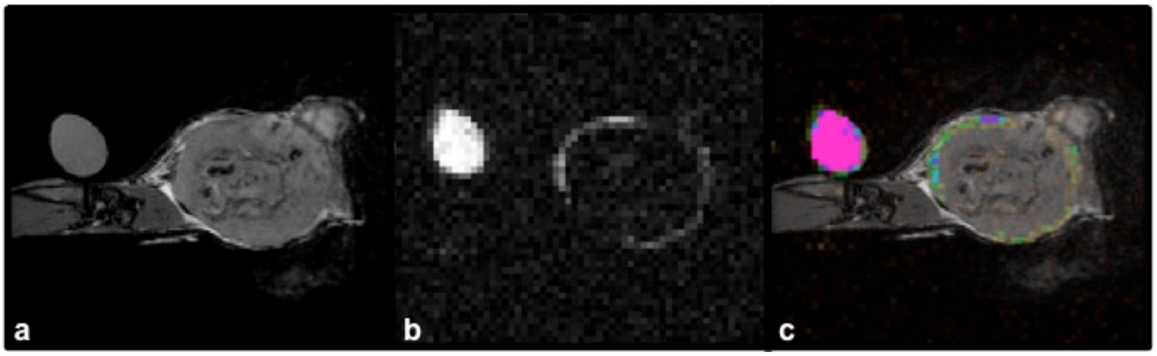

Supplementary Figure S1. ^{19}F-based MRI acquisitions. bSSFP images of mammary fat pad tumors acquired 3 weeks post 4T1 cancer cell implantation, 48 hours after PFC administration, (a) Hydrogen (anatomical), (b) Fluorine-19 and (c) ^{1}H/^{19}F Overlay.
